# Fibroinflammatory Signatures Increase with Age in the Human Ovary and Follicular Fluid

**DOI:** 10.3390/ijms22094902

**Published:** 2021-05-05

**Authors:** Jordan H. Machlin, Seth J. Barishansky, John Kelsh, Megan J. Larmore, Brian W. Johnson, Michele T. Pritchard, Mary Ellen Pavone, Francesca E. Duncan

**Affiliations:** 1 Department of Obstetrics and Gynecology, Feinberg School of Medicine, Northwestern University, Chicago, IL 60611, USA; machlinj@umich.edu (J.H.M.); Sbarishansky@gwu.edu (S.J.B.); MaryEllen.Pavone@nm.org (M.E.P.); 2 Department of Anatomy and Cell Biology, University of Kansas Medical Center, Kansas City, KS 66160, USA; jkelsh@som.umaryland.edu; 3 Department of Comparative Medicine, Histology and Imaging Core, University of Washington, Seattle, WA 98195, USA; larmore@uw.edu (M.J.L.); brianj18@uw.edu (B.W.J.); 4 Department of Pharmacology, Toxicology, & Therapeutics, University of Kansas Medical Center, Kansas City, KS 66160, USA; mpritchard@kumc.edu

**Keywords:** cytokine, fibrosis, inflammation, human, ovary, TGFβ3

## Abstract

The female reproductive system ages before any other organ system in the body. This phenomenon can have tangible clinical implications leading to infertility, miscarriages, birth defects and systemic deterioration due to estrogen loss. “Fibroinflammation” is a hallmark of aging tissues; there is an increase in inflammatory cytokines and fibrotic tissue in the aging ovarian stroma. We systematically evaluated immunomodulatory factors in human follicular fluid, which, like the stroma, is a critical ovarian microenvironment directly influencing the oocyte. Using a cytokine antibody array, we identified a unique fibroinflammatory cytokine signature in follicular fluid across an aging series of women (27.7–44.8 years). This signature (IL-3, IL-7, IL-15, TGFβ1, TGFβ3 and MIP-1) increased with chronologic age, was inversely correlated to anti-Müllerian hormone (AMH) levels, and was independent of body mass index (BMI). We focused on one specific protein, TGFβ3, for further validation. By investigating this cytokine in human cumulus cells and ovarian tissue, we found that the age-dependent increase in TGFβ3 expression was unique to the ovarian stroma but not other ovarian sub-compartments. This study broadens our understanding of inflammaging in the female reproductive system and provides a defined fibroinflammatory aging signature in follicular fluid and molecular targets in the ovary with potential clinical utility.

## 1. Introduction

Aging is associated with a general decline of tissue and cellular function [1]. The reproductive system is unique in that it is the first organ system in the female body to exhibit overt signs of aging. Ovarian function ceases at menopause, which occurs at ~51 years of age [2]. However, even before this, fertility decreases when women are in their mid-30 s due to a decline in egg quantity and quality [2]. Female reproductive aging has important clinical implications because it can lead to infertility, miscarriages, birth defects and systemic deterioration due to reduced estrogen [3]. Evidence from assisted reproductive technology (ART) cycles strongly suggests that the age-dependent decrease in fertility is largely due to defects at the level of the egg. For example, if women use their own eggs to conceive, there is a strong maternal age effect with the likelihood of women in their mid-forties having a live birth being close to zero percent [4]. However, if women use donor eggs from young, healthy women to conceive, the maternal age effect is essentially abrogated [5]. In fact, there are significant age-related changes in the female mammalian gamete, including defects in chromosome structure and cohesion, microtubule and kinetochore function, mitochondrial function and bioenergetics, nucleolar architecture and ribosome numbers, inorganic signaling, gene expression, and epigenetics [6,7,8,9]. However, whether these changes are inherent to the gamete or due to indirect effects of the aging microenvironment are not known.

Fibroinflammation is a hallmark of aging tissues [10,11]. Inflammation is a natural immune response to defend against pathogens or sterile injury, is typically short-lived, and ultimately resolves [12]. However, if acute inflammation persists and becomes chronic, tissue damage and disease pathogenesis can ensue [13]. Chronic inflammation causes fibrosis in which normal wound healing processes are skewed towards overproduction of extracellular matrix and accumulation of scar tissue [14]. We and others have recently demonstrated that the aging ovarian microenvironment or stroma becomes inflammatory and fibrotic with age [15,16]. Reproductive aging in the ovary is associated with increased production and secretion of proinflammatory and pro-fibrotic cytokines and growth factors and a shift in macrophage populations to multinucleated macrophage giant cells [15,17]. In some mouse strains, this inflammation is accompanied by fibrosis characterized by increased collagen I and III levels in mice of advanced reproductive age [15]. Fibrosis and additional changes in the extracellular matrix are also conserved in the aging human ovary, demonstrating the translational relevance of this aging phenomenon [16,18,19].

Although these prior studies focused on the inflammatory and fibrotic nature of the aging ovarian stroma, another critical ovarian microenvironment is the follicular fluid (FF). FF directly surrounds the oocyte as it grows and matures within the cumulus oocyte complex of the antral follicle. The FF is derived from blood plasma macromolecules flowing through the thecal capillaries that cross the blood-follicle barrier and from granulosa and thecal cell secretions themselves [20,21,22]. FF consists of large polysaccharides, hormones, inflammatory factors, reactive oxygen species, and growth factors [21,23]. FF plays a critical role in the development and the eventual quality of the oocyte, and its composition likely reflects the larger circulating biochemical environment [22]. Additionally, the cytokine composition of FF has been shown to vary with age [24].

Given the age-dependent increase in inflammation and fibrosis in the ovarian stroma, we hypothesized that the follicular fluid milieu also becomes fibroinflammatory with age. To test this hypothesis, we used cytokine antibody arrays to semiquantitatively profile the expression of 80 fibroinflammatory cytokines in human follicular fluid from women across an aging series (27.7–44.8 years). We identified a cytokine signature (IL-3, IL-7, IL-15, TGFβ1, TGFβ3 and MIP-1) that increases with chronologic age is inversely correlated to anti-Müllerian hormone (AMH) levels and is independent of body mass index (BMI). Thus, this represents a unique and specific fibroinflammatory reproductive aging cytokine profile in human follicular fluid. We then performed a comprehensive analysis of one fibroinflammatory factor, TGFβ3, in human cumulus cells and ovarian tissue. We found that it increased specifically in the ovarian stroma with age, which may suggest a source for its corresponding increased levels in follicular fluid. These findings advance our understanding of how the human ovarian microenvironment—both the follicular fluid and stromal milieu—change with age and may impact the gametes and follicles that grow within it.

## 2. Results

### 2.1. Chronologic and Reproductive Age Are Associated with an Increase in Specific Subsets of Fibroinflammatory Cytokines

**Figure 1:**
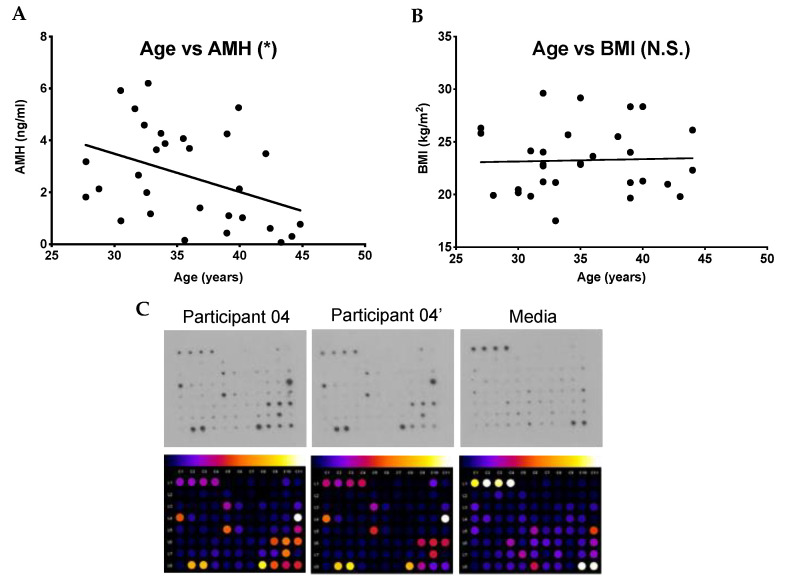
Relationship between age and anti-Müllerian hormone (AMH) and body mass index (BMI) and sample cytokine antibody array (**A**) Graph demonstrating that with increasing age, there was a statistically significant inverse correlation with AMH in our sample population (total *n* = 30). (**B**) Graph demonstrating that with increasing age, there was no statistically significant correlation with BMI in our population. (**C**) Representative human cytokine array C5 performed on the follicular fluid from the first follicle aspirated from either the right (participant 04) or left ovary (participant 04′) from a single participant. One array was incubated in media alone to determine the background and thus, establish signal thresholds. The lines in (**A**) and (**B**) correspond to the fitted regression equation. * *p* < 0.05, N.S. = no statistical significance.

To examine whether fibroinflammatory cytokines increase in human follicular fluid with age, we obtained samples from 30 participants undergoing oocyte aspiration for ART. We interrogated aging from two perspectives: chronologic (i.e., biologic age) as well as reproductive age. These women ranged in age from 27.7 years to 44.8 years (average: 35.7 ± 0.9 y) (Table 1). Anti-Müllerian hormone (AMH) is a hormone produced by growing follicles and is often used as an indirect marker of the ovarian reserve and thus a marker of reproductive age [25,26,27]. In our study, population, serum AMH levels ranged from 0.1 ng/mL to 6.2 ng/mL (average: 2.6 ± 0.33 ng/mL) (Table 1). As expected, because follicle number decreases with age, there was an inverse correlation between age and AMH (Figure 1A) (r^2^ = 0.16, *p* = 0.03). Given that we excluded participants with a BMI ≥ 30 kg/m^2^, the BMI in our participants ranged from 17.5 kg/m^2^ to 29.6 kg/m^2^ (average 23.3 ± 0.57 kg/m^2^) (Table 1). There was no correlation between age and BMI in this population (Figure 1B) (r^2^ = 0.001, *p* = 0.86).

**Figure 2:**
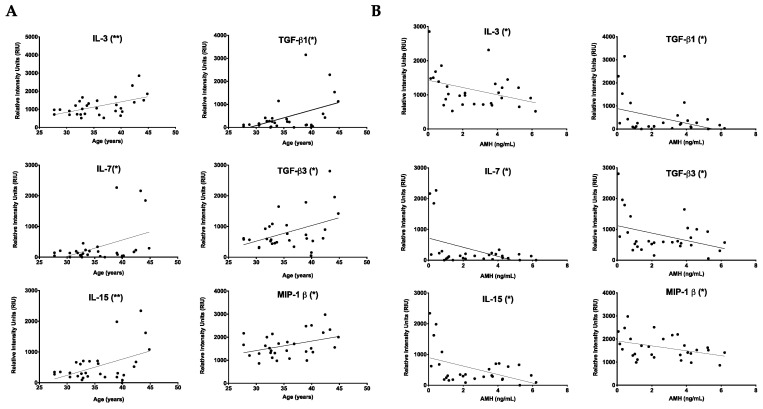
A unique subset of fibroinflammatory cytokines tightly associated with reproductive and chronological aging. (**A**) Graphs showing relative cytokine intensities in the follicular fluid of the first follicle in women undergoing assisted reproductive technology (ART) vs. age. Fibroinflammatory markers IL-3, IL-7, IL-15, TGF-β1, TGF-β3 and MIP-1β were significantly correlated with increasing chronological age (years). (**B**) Graphs showing cytokine markers IL-3, IL-7, IL-15, TGF-β1, TGF-β3 and MIP-1β were also significantly negatively correlated with anti-Müllerian hormone (ng/mL) levels, an indicator for decreased ovarian reserve. The lines correspond to the fitted regression equation. * *p* < 0.05, ** *p* < 0.01.

Using the follicular fluid samples from these participants, we performed an antibody array that allowed the semiquantitative detection of 80 cytokines involved in fibroinflammatory pathways (Figure 1C, Supplemental Figure S2). Of the cytokines interrogated, 61 had levels above a background intensity threshold. To determine whether there was a relationship between these cytokine levels and age, we plotted the intensity values versus chronologic age (years) and reproductive age (AMH). Six cytokines, IL-3 (*p* = 0.0022), IL-7 (*p* = 0.0170), IL-15 (*p* = 0.0084), TGFβ1 (*p* = 0.0108), TGFβ3 (*p* = 0.0193), and MIP-1β (*p* = 0.0315), all showed a positive correlation with chronologic age (Figure 2A). Conversely, the same cytokines were negatively correlated with AMH: IL-3 (*p* = 0.0457), IL-7 (*p* = 0.0154), IL-15 (*p* = 0.0127), TGF-β1 (*p* = 0.0296), TGF-β3 (*p* = 0.0461), and MIP-1β (*p* = 0.0413) (Figure 2B). However, these cytokines did not show any association with BMI (Supplemental Figure S3). Thus, these results demonstrate a unique subset of cytokines that are tightly associated with advanced age defined in two different ways (Table 2). An additional subset of 16 cytokines exhibited a positive correlation with age but not AMH (Table 2, Supplemental Figure S4, and data not shown). The remaining 39 cytokines were not correlated with age (Table 2 and Supplemental Figure S5). Of note, there were no cytokines that showed a significant correlation with AMH and not age (data not shown).

### 2.2. Cytokines That Increase with BMI Are Unique from Those That Change with Age

**Figure 3:**
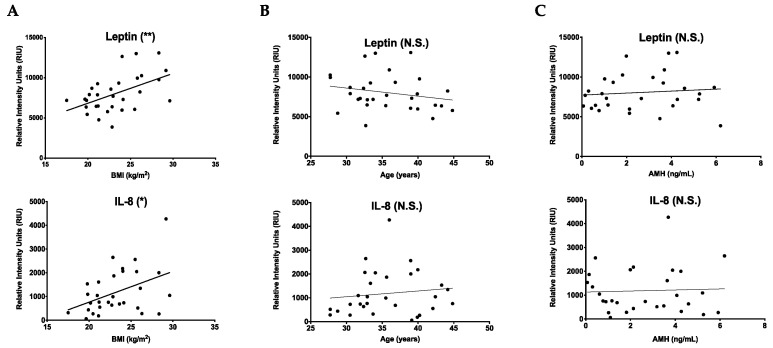
Cytokines that increase with BMI are unique from those that change with age. (**A**) Graphs showing that cytokines leptin and IL-8 were significantly correlated with increasing BMI (kg/m^2^). (**B**) Graphs showing that leptin and IL-8 did not significantly change with increasing age. (**C**) Graphs showing that leptin and IL-8 did not significantly change with increasing AMH. The lines correspond to the fitted regression equation. * *p* < 0.05, ** *p* < 0.01, N.S. = no statistical significance.

Although we selected an upper BMI limit of ≤ 30 kg/m^2^ to avoid confounding effects of obesity on the fibroinflammatory profile in human follicular fluid, there were two cytokines, leptin (*p* = 0.0048) and IL-8 (*p* = 0.0173), that nevertheless were positively correlated with BMI in our sample population (Figure 3A). These cytokines did not correlate with age or AMH (Figure 3B,C). These results validate the efficacy of our cytokine antibody array approach because leptin was previously shown to increase follicular fluid with BMI [28,29]. Moreover, these results demonstrate that the cytokine signature that changes with age is independent of that related to BMI.

### 2.3. TGFβ3 Content in Human Cumulus Cells Does Not Increase with Age

To validate and extend our findings in human follicular fluid, we prioritized a more in depth analysis of TGFβ3, since knowledge of this specific TGFβ isoform is lacking in the human ovary, especially in the context of aging. Transforming growth factor-beta (TGFβ) is a family of proteins with unique immunoregulatory and fibrosis-fostering properties, and in the human, there are three isoforms: TGFβ-1, 2, and 3 [30]. Normally the actions of TGFβ cytokines contribute to tissue repair, and TGFβ action is shut down through regulatory mechanisms that feedback to limit excess TGFβ signaling. However, TGFβ signaling resulting in excessive ECM accumulation can cause fibrosis and scarring [31,32]. Several pathologies stem from TGFβ dysregulation in various organs, including kidney disease, lung, liver and cardiac fibrosis and dermal scarring [31,33]. TGFβ dysregulation has been implicated in female reproductive system pathologies, including PCOS and the pathogenesis of endometriosis and uterine fibroids [32,34].

**Figure 4:**
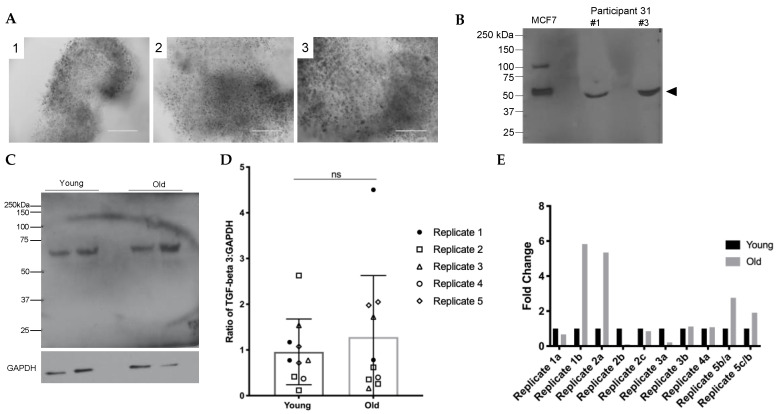
Specific detection of TGFβ3 in human cumulus cells shows no significant difference with age. (**A**) Three representative images of the varying sizes of cumulus cell masses from smaller to larger are shown from participant 42 (39 y). All cumulus cell images were taken upon receipt to the lab. (**B**) We observed a specific immunoreactive band for TGFβ3 at 50 kDa representing the pro-form of the TGFβ3 molecule. MCF7 is the positive control. Two intact cumulus cell masses from participant 31 are shown in lanes 2 and 3. The arrow indicates TGFβ3 (50 kDa). (**C**) Representative blot probed with antibodies recognizing TGFβ3 and reprobed for GAPDH (36 kDa). (**D**) Ratio of TGFβ3 to GAPDH in young and old cumulus cells for all replicates. Young and old groups N.S. (**E**) Fold-change of TGFβ3 in old cumulus cells compared to young cumulus cells for all replicates. More detailed information about each participant can be found in Supplementary Table S1. Scale bar = 100 µm.

Components of human follicular fluid are derived in part from granulosa cell secretions [22]. Therefore, to determine if the increase in TGFβ3 observed in follicular fluid was paralleled in the somatic compartment of the follicle, we performed immunoblot analysis on intact cumulus cell masses (Figure 4A). As a positive control to validate the antibody, we ran protein extracts from MCF7 cells, a hormone-responsive breast cancer cell line known to express TGFβ3, alongside protein extracts from human cumulus cells [35]. Like the control, TGFβ3 was detected as a single immunoreactive band at 50 kDa in whole cumulus cell masses (Figure 4B). To examine whether there were age-dependent changes in TGFβ3 expression in the cumulus cells, we performed immunoblot analysis on protein extracts from cumulus cell masses from reproductively young (<35 y) and old (≥35 y) participants (Figure 4C–E). To minimize confounding variables, we analyzed cumulus cells from reproductively young and old participants that surrounded oocytes that produced the same embryo outcomes on day 5 or day 6, indicating similar quality (Table 3). Densitometry was performed to determine band intensities of the young and old age cohorts, and we normalized TGFβ3 expression to GAPDH. Although the ratio of TGFβ3:GAPDH tended to increase with age, this was not significant (Figure 4D). Moreover, the fold change of TGFβ3 expression between reproductively young and old samples was not consistent across experimental replicates, suggesting that there was no age-dependent change in cumulus cell expression of TGFβ3 (Figure 4E).

### 2.4. TGFβ3 Is Expressed in Follicles, Vasculature and Stroma in the Human Ovary

**Figure 5:**
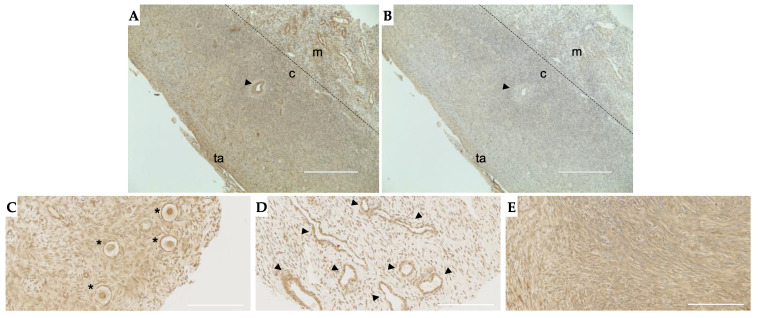
TGFβ3 is found throughout the ovarian stroma, within the follicles and around blood vessels. (**A**) Tissue section with specific staining throughout the stroma and around key structures in the ovary. (**B**) IgG negative control; (**C**) primordial follicles (asterisks); (**D**) blood vessels (arrowheads); (**E**) dense stroma. Panels (**A**,**B**) are representative histological sections performed in *n* = 6 patients, and (**C**–**E**) are cores from the TMA with representative images shown. Arrowheads: blood vessels, ta: tunica albuginea, c: cortex, m: medulla, asterisk: primordial follicles. (**A**,**B**) Scale bar = 400 µm; (**C**,**D**) scale bar = 100 µm.

Although we did not see age-associated changes in TGFβ3 expression in cumulus cells, it is possible that its expression increased in the ovarian microenvironment especially given that TGFβs are anchored in the extracellular matrix (ECM) in a latent form. Thus, we next examined TGFβ3 localization in the human ovary via immunohistochemistry on tissue sections. TGFβ3 expression was observed throughout the ovarian stroma with more dense staining at the cortex than the medulla (Figure 5A). This staining, except for that at the tunica albuginea, was attenuated when the tissue was stained with the same concentration of nonimmune IgG, confirming specificity (Figure 5B). A further examination of sub-compartments within the ovary demonstrated that TGFβ3 is specifically expressed in follicles, surrounding the vasculature, and throughout the stroma (Figure 5C–E).

### 2.5. Stromal and Vascular TGFβ3 Content Increases with Age in the Human Ovary

**Figure 6:**
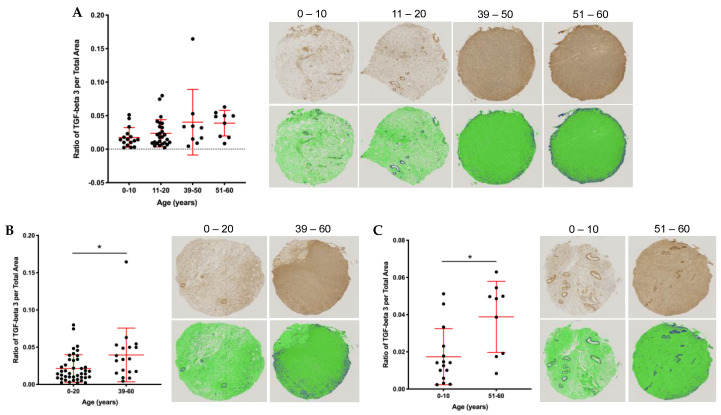
Quantitative analysis of human ovarian tissue microarray (TMA) reveals increased TGFβ3 content with age. (**A**) Average ratio of TGFβ3 expression per total area for core 1 and core 2 (unique cores from the same patient) broken down into four age cohorts: 0–10, 11–20, 39–50, 51–60. Images from cores closest to the average data point in each age cohort are shown with TGFβ3 staining (brown) in the panel above and quantitative data analysis in green and blue below. The dark blue area represents a positive TGFβ3 signal above the threshold, while green is TGFβ3 negative. (**B**) Ratio of TGFβ3 per total area in the 0–20 vs. 39–60 age cohorts. (**C**) Ratio of TGFβ3 per total area in the 0–10 vs. 51–60 age cohorts. Asterisks (*) indicate statistical significance with panel (**B**) *p* = 0.01 and panel (**C**) *p* = 0.005.

To investigate age-associated differences in ovarian TGFβ3 expression, we performed immunohistochemistry using a TMA containing cortical tissue from 60 participants in two age cohorts: 1–20 y and 39–58 y [16]. (Supplemental Figure S1). TGFβ3 staining qualitatively appeared more intense in the samples from the older cohort, so we performed further quantification (Supplemental Figure S1B). First, we created two additional cohorts and thus quantified expression in four age cohorts across an aging continuum: 0–10, 11–20, 39–50, 51–60 (years) using an average of both the core 1 and core 2 data per participant (Figure 6A). There was a trend towards increased TGFβ3 expression across these four age cohorts (Figure 6A). This increased expression was significant when comparing TGFβ3 content between the 0–20 y and 39–60 y (*p* = 0.012) and 0–10 y and 51–60 y (*p* = 0.006) cohorts (Figure 6B).

**Figure 7:**
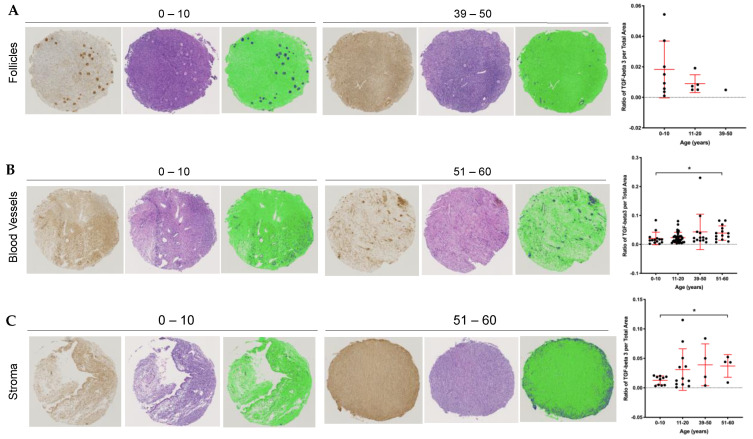
Analysis of ovarian cores from the TMA reveal changes in TGFβ3 content with age depending on the presence of landmark tissue structures. Each core was categorized into one of three major tissue structures: follicles, blood vessels or stroma. Representative images of youngest and oldest age cohort’s cores are shown with the first panel: TGFβ3 staining in the brown, middle panel: hematoxylin and eosin stain and last panel: quantitative data measurement with positive TGFβ3 signal in dark blue and negative TGFβ3 stain in green. (**A**) Average core 1 and 2 ratios of TGFβ3 per total area across age cohorts with follicles. (**B**) Average core 1 and 2 ratios of TGFβ3 per total area across age cohorts with blood vessels. (**C**) Average core 1 and 2 ratios of TGFβ3 per total area across age cohorts with stroma. Asterisks (*) indicate statistical significance with panel (**B**) *p* = 0.04 and panel (**C**) *p* = 0.004.

Although each of the cores from the TMA was obtained from the pathologist-confirmed ovarian cortex, there was heterogeneity in the tissue cores. Some cores contained follicles, others had blood vessels, and there were varying amounts of stroma throughout. We, therefore, analyzed TGFβ3 content taking into account the presence of dominant structures within the tissue. Based on H&E staining, cores were classified into those containing follicles, blood vessels, or stroma. We plotted the average TGFβ3 per total area across the same four age cohorts, stratified by the three ovarian structures (Figure 7). Cores containing follicles showed a trend towards a decrease in TGFβ3 levels with age, but this analysis is limited by the age-dependent decrease in the number of cores containing follicles (Figure 7A). In contrast, cores containing blood vessels and stroma demonstrated increased TGFβ3 content with age when comparing the 0–10 y vs. 51–60 y cohorts (Figure 7B,C, *p* = 0.042 blood vessels and *p* = 0.004 stroma). Therefore, the age-associated increase in TGFβ3 expression in the ovary occurs in tissue regions containing stroma and vasculature.

## 3. Discussion

In this study we interrogated the fibroinflammatory signature of human follicular fluid. Both maternal age and elevated BMI can negatively impact fertility and affect the circulating levels of cytokines, but the precise mechanisms by which these factors impact oocyte quality are not fully elucidated [36,37,38,39,40,41,42,43]. We identified six cytokines, IL-3, IL-7, IL-15, TGFβ1, TGFβ3, and MIP-1, which were positively correlated with chronologic age and inversely correlated with AMH, an indirect marker of the ovarian reserve or reproductive age [10]. These specific cytokines, however, were not correlated with BMI. Obesity alters the levels of certain adipokines, and although there was an upper BMI limit of ≤30 kg/m^2^ in our study, we identified leptin and IL-8 as being positively correlated with BMI. These findings are consistent with others, demonstrating that leptin is a principal cytokine that increases with BMI in human follicular fluid [28,29]. Leptin is produced by granulosa and cumulus cells of ovarian follicles [44] and can impair follicle development, ovulation, and oocyte maturation [45,46]. Like leptin, IL-8 is also part of the immunologic profile associated with excessive body weight [47]. The observation that IL-8 and leptin levels correlated with BMI, but not age and that IL-3, IL-7, IL-15, TGFβ1, TGFβ3, and MIP-1 levels correlated with age, but not BMI suggest distinct fibroinflammatory responses in obesity and aging, and thereby unique mechanisms by which these factors negatively impact oocyte quality.

There are several potential origins of the unique fibroinflammatory signature seen with aging. These cytokines could be due to the known age-associated shift of macrophage plasticity and ontogeny in the ovary [48]. For example, ovarian aging is associated with increased multinucleated macrophage giant cells, which may be responsible for a highly inflammatory cytokine milieu [15,17]. An alternative possibility is that senescent ovarian cells, which may or may not be immune cells, may produce an inflammatory milieu. “Inflammaging,” a leading theory on aging, refers to the low-grade, chronic inflammation that underlies aging tissues and pathologies in the absence of overt infection [49,50]. Senescent cells, which increase with age, are a key source of inflammaging [51]. Cellular senescence is a process in which cells cease dividing but remain metabolically active and undergo distinctive phenotypic alterations, fueling aging [52]. These changes include secretion of cytokines, chemokines, growth factors, and proteases, which are collectively referred to as the senescence-associated secretory phenotype (SASP) [53].

Our data are consistent with the well-documented SASP in other tissues by way of composition. Specifically, two of the cytokines that we found to increase with age in follicular fluid, IL-7 and IL-15, are well-characterized senescent cell markers [54]. IL-7 is implicated in aging due to its role in the generation and maintenance of naive and memory CD8 and CD4 T cells [55,56]. Increased concentrations of IL-15 in follicular fluid from women undergoing in vitro fertilization are negatively correlated with achieving a clinical pregnancy [57]. MIP-1β, another fibroinflammatory chemokine that increased with age in our samples, is also a potential biomarker for inflammaging [58]. On the other hand, we did not identify an age-dependent increase in other conserved SASP components, including IL-6, IL-8, GROα, or GROβ. This could be because SASP composition is diverse and highly tissue and cell-type-dependent, suggesting that the ovary may have a unique signature [54,59,60]. However, future studies are necessary and ongoing to determine whether senescent cells exist and drive inflammaging in the ovary.

Our results also provide insight into other chronic inflammatory regulators that can induce specific fibrotic pathways, such as those regulated by the transforming growth factor-beta (TGFβ) family. The three TGFβ family isoforms have unique immunoregulatory properties that normally contribute to tissue repair [30]. However, excess action of TGFβ involving surplus ECM deposition can cause fibrosis and scarring [31,32]. This type of pathogenic dysregulation is more widely documented in the TGFβ1 and 2 isoforms [31,32,61]. Interestingly, our results demonstrated that TGFβ3 increased specifically with age, was inversely correlated with AMH, and was independent of BMI. Although TGFβ3 is considered a proliferative growth factor and a scar-preventive protein, there is evidence that excessive TGFβ3 signaling can cause tissue fibrosis and disease progression [62,63,64]. For example, TGFβ3 levels can be elevated 3–5 times more in uterine fibroids than in normal myometrium [62].

In the human ovary, TGFβ3 localized throughout the stroma, blood vessels, and surrounding follicles. These findings are consistent with previous observations in the bovine ovary, where TGFβ isoforms are expressed in follicles and likely act as important paracrine and autocrine signaling molecules regulating ovarian follicle growth and proliferation [65]. Interestingly, TGFβ3 expression decreased with age in histological sections of the ovarian cortex that contained follicles. Since this factor is important for growth and proliferation, a reduced expression with age may be correlated with changes in follicle quality. Finally, we observed increased expression of TGFβ3 with age in tissue sections containing stroma and vasculature but not in the cumulus granulosa cells. The TGFβ family has angiogenic and angiostatic properties depending on expression levels and the tissue context [66]. TGFβ3 may propagate fibroinflammation by increasing vasculature giving rise to additional sources where cytokines and inflammatory molecules may accumulate in the ovary with advancing age. Interestingly, we also saw increased vascular endothelial growth factor (VEGF) expression with age in human follicular fluid. This is consistent with data that women of advanced reproductive age undergoing IVF have higher FF levels of VEGF, which could reflect a hypoxic follicular environment [67]. Thus, increased vasculature and pro-angiogenic factors may act as a compensatory mechanism for promoting blood supply and oxygen through the dense fibrotic tissue.

Overall, our findings advance our knowledge of age-related inflammation in the ovary by defining a unique subset of fibroinflammatory cytokines associated with aging. Whether these cytokines are a cause or consequence of female reproductive aging is not known. The TGFβ pathway is currently being investigated as a therapeutic target for pathologies in the reproductive system, and our findings suggest that these strategies may also have relevance in ovarian aging. However, future studies are needed to decipher the relationship between the TGFβ3 that increases with age in ovarian tissue and in the follicular fluid. Translational studies are also ongoing to determine whether these particular cytokines influence gamete quality and whether these effects are direct on the oocyte or indirect via the supporting somatic cells. The fibroinflammatory cytokine/chemokine signature in the follicular fluid may also be an important biomarker of the quality and developmental potential of the oocyte, which may have predictive value for assisted reproductive technologies outcomes. Moreover, the aging follicular fluid signature may reflect systemic aging, which would broaden our understanding of fertility as a marker of overall health.

## 4. Materials and Methods

### 4.1. Human Follicular Fluid, Cumulus Cells, and Ovarian Tissue

Follicular fluid was obtained from women undergoing oocyte retrieval for a noncancerous diagnosis at Fertility and Reproductive Medicine (FRM) (Northwestern University, Chicago, IL, USA) through the Northwestern University Reproductive Tissue Library (NU-RTL), an Institutional Review Board-approved tissue repository protocol, following written informed consent. Inclusion criteria were women undergoing oocyte retrieval ≥ 18 y, ≤30 kg/m^2^, and English speaking. Participants with multiple controlled ovarian stimulation cycles, a history of reproductive pathologies or other conditions associated with inflammation were excluded. To avoid blood contamination, follicular fluid aspirated only from the first follicle from each ovary was saved from each participant. Upon collection, a Halt protease inhibitor cocktail (Thermo Fisher Scientific, Waltham, MA, USA) was added to the follicular fluid samples to a final concentration of 1×. The follicular fluid was then centrifuged at 300× *g* for 20 min at 4 °C to pellet cellular debris. The supernatant was then transferred to sterile tubes, snap-frozen in liquid nitrogen and stored at −80 °C until utilized in the cytokine antibody arrays. In addition to follicular fluid, participant-specific health information was obtained, including age, body mass index (BMI), and serum levels of anti-Müllerian hormone (AMH) (Table 1).

We also obtained human cumulus cells that would have otherwise been discarded from women undergoing oocyte retrieval at the FRM clinic through the NU-RTL. The inclusion and exclusion criteria were the same as above, but for cumulus cells, participants had to be undergoing intracytoplasmic sperm injection and preimplantation genetic testing. The oocytes retrieved were used clinically, whereas a portion of the cumulus cells from the larger cumulus–oocyte complexes (COC) was designated for research purposes. Each cumulus mass was assigned a number corresponding to the oocyte to track the oocyte and its respective embryo outcome in a one-to-one manner. All cumulus cells were transferred from the clinic in 4-well dishes containing HEPES buffered human tubal fluid (HTF) media (Irvine, CA, USA, Catalog #90,126) and placed in an incubator (37 °C, 5% CO_2_) before processing. Each cumulus cell mass was imaged on an EVOS FL auto microscope (Life Technologies Corporation, Grand Island, NY, USA). Next, cumulus cell masses were rinsed through six large drops of Leibovitz’s L-15 Medium (Thermo Fisher Scientific, Waltham, MA, USA) supplemented with 3 mg/mL polyvinylpyrrolidone (PVP) and 0.5% penicillin–streptomycin (PS) to remove any exogenous protein from the HTF media. Cumulus cells were then snap-frozen in a minimal volume of L-15 medium and stored at −80 °C. Deidentified information for each participant was obtained, including age, diagnosis, cumulus cell number corresponding to the oocyte retrieved, the oocyte’s maturity status and embryo outcome (Supplementary Table S1). We received an average of 7 cumulus cell masses per participant and utilized cumulus cells from a total of 12 participants samples for this study.

De-identified, formalin-fixed, paraffin-embedded (FFPE) human ovarian tissue samples used for this study were obtained through two established tissue archives at Northwestern University: the Oncofertility Consortium’s National Physicians Cooperative (OC-NPC) and the NU-RTL. All procedures for tissue collection were performed under IRB-approved protocols after obtaining informed consent from each patient. Tissues obtained through the OC-NPC were from patients undergoing ovarian tissue cryopreservation for fertility preservation purposes. Under these protocols, 80% of the tissue was cryopreserved for future patient use to restore endocrine function and/or fertility, and 20% was donated to research. A portion (10%) of the research tissue was fixed, paraffin-embedded and stored in a fixed tissue archive, accessed for this study. Tissue blocks were obtained and either used to generate an ovarian tissue microarray (TMA) as described previously or were sectioned at 5 µm thickness for immunohistochemistry as also described below [16].

### 4.2. Cytokine Antibody Arrays

Cytokine antibody arrays were performed on human follicular fluid using the Ray Bio C-series human cytokine antibody array C5 (Ray Biotech Inc, Norcross, GA, USA) according to the manufacturer’s instructions. Human follicular fluid samples from each participant were run in duplicate, and 500 μL of follicular fluid was used on each array. Although media contamination in the follicular fluid samples was anticipated to be low because only the first follicle from each ovary was used, we performed cytokine antibody arrays on media alone to determine the background and, thus, establish signal thresholds. The cytokine antibody arrays were visualized using chemiluminescence according to the manufacturer’s instructions. The cytokine antibody arrays were imaged, and the Protein Array Analyzer Plugin for ImageJ was used to quantify signal intensity using densitometry [68]. The region of interest for each cytokine measured was kept constant across all experiments. Intensity values were exported into Ray Biotech’s analysis tool, and intensity values were normalized to the oldest participant. Relative intensity values for each cytokine were plotted versus age, BMI, and AMH values.

### 4.3. Antibodies

To detect TGFβ3, we used a rabbit polyclonal antibody (Abcam, AB15537, Cambridge, MA, USA) that recognizes the pro-form of the TGFβ3 molecule (50 kDa) for immunohistochemistry at a 1:100 dilution (0.01 µg/µL) and immunoblot analysis at a 1:500 dilution (0.002 µg/µL). A nonimmune rabbit IgG antibody (Vector Laboratories, Inc, Burlingame, CA, USA) was used as a negative control at the same concentrations as the TGFβ3 antibody. We used a rabbit monoclonal antibody against GAPDH (36 kDa) (Catalog 5174S, Cell Signaling Technology, Danvers, MA, USA) at a 1:2000 dilution as a protein loading control to normalize TGFβ3 immunoreactivity in immunoblots. A horseradish peroxidase-conjugated donkey anti-rabbit secondary antibody was purchased from GE Healthcare Life Sciences (Pittsburgh, PA, USA) and was used to detect both primary antibodies in the immunohistochemistry and immunoblot analyses.

### 4.4. Protein Extraction and Immunoblot Analysis (Non-Reducing Conditions)

Cumulus cells were thawed in a 1× cell lysis buffer (Cell Signaling Technology, Danvers, MA, USA) supplemented with Halt™ Protease and Phosphatase inhibitor Cocktail (100x) (Thermo Fischer Scientific, Waltham, MA, USA). Cells were homogenized using a Pellet Pestles cordless motor (Sigma Aldrich, St. Louis, MO, USA) for 30 s intervals and spun down for 20 min at 10,000 g at 4 °C. The supernatant was divided into 5 µL aliquots, which were either used immediately for immunoblot analysis or stored at −80 °C. To run immunoblots, cumulus cell protein extract (run individually with one extract from one cumulus cell per lane) and molecular weight standards (Bio-Rad, Hercules, CA, USA) were loaded onto precast 10% MiniPROTEAN TGX gels (Bio-Rad, Hercules, CA, USA) and transferred onto 0.45 µm PVDF membranes (GE Healthcare Life Sciences). All steps were done at room temperature unless otherwise specified. Membranes were blocked with 3% ECL Blocking Agent (GE Healthcare Life Sciences) in 1× Tris-buffered saline (TBS; 20 mM Tris-HCL and 150 mM NaCl (pH 7.6)) containing 0.1% Tween-20 (TBS-T) for 3–4 h. Blots were incubated overnight in primary antibodies (TGFβ3 and GAPDH) diluted in a 3% ECL block at 4 °C. Blots were rinsed three times with 1× TBS-T for 3 × 30 min and incubated with secondary antibody diluted in 3% ECL block for 1 h. After rinsing three times again with 1× TBS-T for 30 min each, blots were developed using GE Healthcare Amersham ECL detection reagent. Blots were then reprobed with an antibody against GAPDH, which served as a loading control. To do this, blots were rinsed in 1× TBS-T and stripped in 5 mL of Restore stripping buffer (ThermoFisher, Waltham, MA, USA) for 15 min, rinsed 4 × 5 min in 1× TBS-T, and processed as described above for the TGFβ3 antibody. Films were scanned, and densitometry was performed using ImageJ software (National Institutes of Health, Bethesda, MD, USA). Individual band intensities were measured and normalized to the GAPDH band intensities. All graphs were created using GraphPad Prism 7 software.

### 4.5. Immunohistochemistry (IHC)

To detect TGFβ3 in human ovarian tissue, we performed IHC on whole ovarian tissue sections and on TMAs. Slides containing human FFPE ovarian tissue sections were deparaffinized in Citrisolv (Thermo Fisher Scientific, Waltham, MA, USA) and rehydrated through a graded ethanol series (100% EtOH 2 × 3 min, 95% EtOH 1 × 3 min, 85% EtOH 1 × 3 min, 70% EtOH 1 × 3 min, 50% EtOH 1 × 3 min ddH_2_O 1 × 3 min). Slides were rinsed in TBS-T and then incubated in 3% H_2_O_2_ for 15 min to quench endogenous peroxidases. A Pap pen (Vector Laboratories, Inc., Burlingame, CA, USA) was used to experimental and control sections on each slide. Slides were rinsed in 1× TBS, and endogenous biotin and avidin binding was blocked with an avidin/biotin blocking kit (Vector Laboratories, Inc., Burlingame, CA, USA). Nonspecific binding was attenuated by incubating slides in blocking solution according to the VECTASTAIN Elite ABC System (Vector Laboratories, Inc., Burlingame, CA, USA) with 1× TBS as the buffer. Experimental slide sections were incubated with the primary antibody, and control sections were incubated in nonimmune rabbit IgG overnight at 4 °C. Slides were rinsed again in TBS-T and incubated in biotinylated antibody according to manufacturer’s instructions (VECTASTAIN Elite ABC System, Vector Laboratories, Inc., Burlingame, CA, USA) for two hours at room temperature. Slides were washed in TBS-T and incubated in an ABC reagent (Vector Laboratories, Inc., Burlingame, CA, USA) for 30 min at room temperature. Slides were then washed again in TBS-T, and staining was visualized using the DAB peroxidase (HRP) substrate kit (Vector Laboratories, Inc., Burlingame, CA, USA), which resulted in a brown precipitate. The DAB reaction was stopped by placing slides in dH_2_O for 5 min as soon as the signal was first visible in the negative control section. Slides were then counterstained in hematoxylin (Harris Hematoxylin, Mercedes Medical, Sarasota, FL, USA) and then coverslipped with Cytoseal XYL (Thermo Fisher Scientific, Waltham, MA, USA). Stained slides were imaged on an EVOS FL auto microscope (Life Technologies Corporation).

### 4.6. Human Ovarian Tissue Microarray (TMA) TGFβ3 Analysis

We used a previously generated human ovarian TMA that included FFPE tissue blocks from 60 participants ranging from 0–20 and ≥39 [16]. The cores containing all 60 samples were arranged in duplicate on the TMA block (Supplemental Figure S1). Samples from each tissue archive were randomly distributed concerning age in the TMA block to avoid staining bias. Cores from the human liver (fibrotic and non-fibrotic) and ovarian cancer tissues were included as controls. IHC for TGFβ3 was performed on a TMA section as described above. Due to the precious nature of the human ovarian tissue, we did not want to apply a nonimmune IgG on one of the TMA slides as a negative control. Instead, as a positive control and an indicator for stopping the DAB reaction, we ran a slide containing human ovarian tissue in parallel with the TMA section. Additionally, a sequential TMA section was stained with hematoxylin and eosin (H&E) according to the standard procedure to correlate TGFβ3 expression with specific histological structures in the ovarian cores.

To quantify TGFβ3 expression in each core, whole slides were scanned in bright-field using a 20X objective on a NanoZoomer digital pathology system (Hamamatsu City, Japan) at the University of Washington Histology and Imaging Core (Seattle, Washington, DC, USA). The digital images were then imported into Visiopharm software (Hørsholm, Denmark) for analysis. Using the Visiopharm image analysis module, regions of interest (ROI) were manually drawn around each TMA core. The digital images were converted into grayscale values using two-color space bands, FastRed_DAB—DAB and RGB—B, with the mean filter of 3 × 3 pixels. The software training to determine positive TGFβ3 staining and background tissue counterstain (hematoxylin) was performed on three ROIs from two serial sections of control tissue staining in conjunction with the TMA slides. From the control slide, one section was stained with TGFβ3 and the other with IgG isotype-matched control. To be conservative in our thresholding, we averaged three different ROIs from the IgG isotype-matched control. These represented areas of low, medium and high background intensity (Supplemental Figure S1D). Individual thresholds of pixel values were created for each of the three ROIs, averaged to account for tissue heterogeneity and then the average threshold was applied to the TMA for analysis. The TMA cores were processed in batch mode using this configuration to generate the desired per area outputs and analyzed at 100%. Based on this strategy, the green label visible in the images corresponds to the thresholding for tissue that is not considered positively stained, whereas the blue label is any tissue considered positively stained. Once the analysis was complete, we received a spreadsheet indicating the ratio of positive TGFβ3 staining per total core area for each core run in duplicate. These values were used for all quantification and graphical analysis on the TMA.

To stratify the patterns of TGFβ3 localization based on the presence of specific cellular features within each tissue core, we analyzed the sequential section of the TMA that was H&E-stained. We classified all 120 cores as being enriched in either follicles, blood vessels, or stroma. If any follicle was present, the core was classified as the follicle. The vasculature category was assigned if 3 or more medium (>75 µm) to large blood vessels were present. If most of the tissue (~50% or greater) was stroma and it lacked a follicle, then the core was classified as stroma. We then stratified the ratio of TGFβ3 expression per core area by specific structure-based categories or “landmark features.”

### 4.7. Statistical Analysis

To examine the relationship between maternal age, AMH, BMI and cytokine relative intensities in follicular fluid, simple linear regression analysis (Pearson’s coefficient) was used. Goodness-of-fit was quantified using R-squared and sum-of-squares. Statistically significant differences between two independent groups were analyzed using unpaired t-test, and comparisons of more than two independent groups were analyzed using one-way analysis of variance (ANOVA) followed by multiple comparisons test (Tukey post hoc test). *p* < 0.05 was considered statistically significant. Statistical analysis was performed using GraphPad Prism Version 8.1.2 software (GraphPad Software Inc., La Jolla, CA, USA).

## Figures and Tables

**Table 1:** Participant-specific health information.

Participant ID	Age (Years)	AMH (ng/mL)	BMI (kg/m^2^)
Participant 01	27.7	1.8	26.3
Participant 02	27.7	3.2	25.8
Participant 03	28.8	2.1	19.9
Participant 04	30.5	5.9	20.5
Participant 05	30.5	0.9	20.2
Participant 06	31.6	5.2	19.8
Participant 07	31.9	2.7	24.1
Participant 08	32.4	4.6	22.7
Participant 09	32.6	2.0	24.0
Participant 10	32.7	6.2	22.9
Participant 11	32.9	1.2	21.2
Participant 12	32.9	1.9	29.6
Participant 13	33.6	3.6	21.1
Participant 14	33.7	4.3	17.5
Participant 15	34.0	3.9	25.7
Participant 16	35.5	4.1	22.9
Participant 17	35.6	0.2	23.0
Participant 18	36.0	3.7	29.2
Participant 19	36.8	1.4	23.6
Participant 20	39.0	0.4	25.5
Participant 21	39.0	4.3	28.3
Participant 22	39.1	1.1	19.7
Participant 23	39.9	5.3	21.1
Participant 24	40.0	2.1	24.0
Participant 25	40.2	1.0	28.4
Participant 26	42.1	3.5	21.3
Participant 27	42.4	0.6	21.3
Participant 28	43.3	0.1	21.0
Participant 29	44.2	0.3	26.1
Participant 30	44.8	0.8	22.3
Average (± SE)	35.7 ± 0.90	2.6 ± 0.33	23.3 ± 0.57

**Table 2:** Relationship between fibroinflammatory cytokines and biologic and reproductive aging.

Parameters	Fibroinflammatory Cytokines
Age and AMH	IL-3, IL-7, IL-15, MIP-1β, TGF-β1, TGF-β3
Age	M-CSF, SDF-1, EGF, oncostatin M, VEGF, PDGF-BB, BLC, CK β 8-1, BDNF, FGF-9, GDNF, IGFBP-3, LIF, MIF, PIGF, TGF-β2
Non-significant	GRO, GRO-α, IL-β, IL-8, IL-12 p40, MCP-1, MCP-2, MCP-3, MIP-1δ, RANTES, SCF, IGF-1, angiogenin, leptin, eotaxin 1, eotaxin 2, eotaxin 3, FGF-4, FGF-6, FGF-7, Flt-3 ligand, fractalkine, GCP-2, HGF, IGFBP-1, IGFBP-2, IGFBP-4, IL-16, IP-10, LIGHT, MIP-3α, NAP-2, NT-3, NT-4, osteopontin, osteoprotegerin, PARC, TIMP-1, TIMP-2

**Table 3:** Young vs. old participant cumulus cell information.

Group	Participant ID	Age	Cumulus Mass ID	Embryo Outcome
1	Participant 32	32.5	a	Blastocyst day 6
			b	Blastocyst day 6
	Participant 33	44.7	a	Blastocyst day 6
			b	Blastocyst day 5
2	Participant 34	33.9	a	Hatching blastocyst day 5
			b	Hatching blastocyst day 5
			c	Hatching blastocyst day 6
	Participant 35	41.5	a	Blastocyst day 5
			b	Blastocyst day 6
			c	Blastocyst day 6
3	Participant 36	34.4	a	Blastocyst day 6
			b	Blastocyst day 6
	Participant 37	42.3	a	6 cell day 6
			b	Mature (not fert.)
4	Participant 38	34.8	a	Blastocyst day 5
	Participant 39	40.1	a	Blastocyst day 5
5	Participant 40	34.9	b	Early blastocyst day 6
			c	Morula day 6
	Participant 41	43	a	10 cell day 6
			b	6 cell day 6

## Data Availability

Data are available from the corresponding author upon reasonable request.

## Supplementary Materials

The following are available online at https://www.mdpi.com/article/10.3390/ijms22094902/s1, Figure S1: Human ovarian tissue microarray (TMA) layout with sample age breakdown and threshold intensity method, Figure S2: RayBio C-series human cytokine antibody array C5 decoder of cytokines in Figure 1C, Figure S3: Cytokine profiles that were significantly different with age or AMH, but did not change with BMI, Figure S4: Follicular fluid cytokine profiles that increased with chronologic age, but not with reproductive age (AMH), Figure S5: Cytokine profiles in the follicular fluid that showed no change with chronologic age, Table S1: Participant cumulus cell information.
