# Supplementary material for: Fibroinflammatory Signatures Increase with Age in the Human Ovary and Follicular Fluid
Source: Int J Mol Sci. 2021 May 5;22(9):4902. doi: 10.3390/ijms22094902 (PMC8125514; doi:10.3390/ijms22094902)
Supplement: Supplementary file 1 [file ijms-22-04902-s001.zip › ijms-1168078-con-sup-update.pdf]

International Journal of Molecular Sciences

MDPI

Supplemental Material

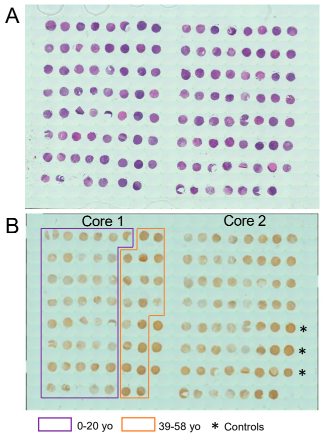

C

TMA Patient Demographic Information

	1	2	3	4	5	6	7	8
1	Age: 16 yrs Non Hodgkin's lymphoma	Age: 13 yrs Aplastic anemia	Age: 16 yrs Low grade midline glioneuronal tumor	Age: 17 yrs Osteosarcoma	Age: 18 yrs Hodgkin Lymphoma	Age: 10 yrs Turner's Syndrome	Age: 48 yrs Leiomyoma	Age: 45 yrs Leiomyoma
2	Age: 16 yrs Ewing Sarcoma	Age: 13 yrs Ewing Sarcoma	Age: 2 yrs * Sickle Cell Anemia	Age: 9 yrs Fanconi's Anemia	Age: 8 Medulloblastoma	Age: 54 yrs Leiomyoma	Age: 44 Leiomyoma	Age: 49 yrs Leiomyoma
3	Age: 15 yrs * Medulloblastoma	Age: 15 yrs Lymphoma	Age: 18 yrs Lymphoma	Age: 19 yrs Ewing Sarcoma	Age: 7 Ewing Sarcoma	Age: 53 yrs Pelvic pain, R cyst	Age: 41 Leiomyoma	Age: 50 AUB
4	Age:17 yrs Lymphoma	Age: 15 yrs Rhabdomyosarcoma	Age: 16 yrs Hodgkin lymphoma	Age: 15 yrs Hodgkin Lymphoma	Age: 11 yrs Sickle Cell Disease	Age: 53 yrs Leiomyoma	Age: 39 Leiomyoma	Age: 51 Leiomyoma
5	Age: 19 yrs Ewing Sarcoma	Age: 13 yrs Ewing Sarcoma	Age: 10 yrs Ewing Sarcoma	Age: 8 yrs Fanconi Anemia	Age: 6 yrs Beta-thalassemia	Age: 52 Leiomyoma	Age: 53 Prolapse	Non-Fibrotic Liver
6	Age: 17 yrs Ewing Sarcoma	Age: 3 yrs Sarcoma	Age: 8 yrs Osteosarcoma	Age: 5 yrs Fanconi Anemia	Age: 4 yrs Clear cell sarcoma of kidney	Age: 52 Leiomyoma	Age: ? Diagnosis ?	Fibrotic liver
7	Age: 17 yrs Hodgkin's lymphoma Stage IV	Age: 9 yrs Ewing Sarcoma	Age: 17 yrs Osteosarcoma	Age: 18 yrs Aplastic Anemia	Age: 18 yrs Pineal Blastoma	Age: 51 Not reported	Age: 53 AUB	Ovarian Cancer
8	Age: 11 yrs Sickle Cell Anemia	Age: 22 mos Rhabdomyosarcoma	Age: 12 yrs Beta-thalassemia Major	Age: 9 yrs Severe Aplastic Anemia	Age: 18 Medulloblastoma	Age: 50 Leiomyoma	Age: 47 Leiomyoma	

* Indicates loose or small tissue noted at time of punch by Path core

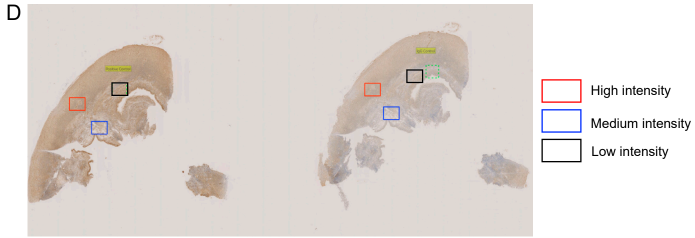

Supplemental Figure S1. **Human Ovarian Tissue Microarray (TMA) layout with sample age breakdown and threshold intensity method. (A)** TMA stained using H&E **(B)** TMA stained using antibodies recognizing TGFβ3 (brown). Purple and orange boxes indicate young and old participant's samples respectively. Asterisks indicate three control samples used which include non-fibrotic liver, fibrotic liver, and ovarian cancer tissues. Core 1 and Core 2 are duplicate patient samples. **(C)** Detailed information about each participant in the TMA **(D)** Thresholding method for each core within the TMA. Three areas of intensity were averaged together from the positive TGFβ3 stained ovary (left) and the negative IgG control ovary (right) to determine background for each core.

	A	B	C	D	E	F	G	H	I	J	K
1	POS	POS	POS	POS	NEG	NEG	ENA-78	G-CSF	GM-CSF	GRO	GRO-α
2	I-309	IL-1α	IL-1β	IL-2	IL-3	IL-4	IL-5	IL-6	IL-7	IL-8	IL-10
3	IL-12 p40/p70	IL-13	IL-15	IFN-γ	MCP-1	MCP-2	MCP-3	M-CSF	MDC	MIG	MIP-1β
4	MIP-1δ	RANTES	SCF	SDF-1	TARC	TGF-β1	TNF-α	TNF-β	EGF	IGF-1	ANG
5	OSM	THPO	VEGF	PDGF BB	Leptin	BDNF	BLC	CCL23	Eotaxin 1	Eotaxin 2	Eotaxin 3
6	FGF 4	FGF 6	FGF 7	FGF 9	Flt-3 Ligand	Fractalkine	GCP-2	GDNF	HGF	IGFBP 1	IGFBP 2
7	IGFBP 3	IGFBP 4	IL-16	IP-10	LIF	LIGHT	MCP-4	MIF	MIP-3α	NAP-2	NT-3
8	NT-4	OPN	OPG	PARC	PLGF	TGF-β2	TGF-β3	TIMP-1	TIMP-2	POS	POS

■

Supplemental Figure S2. RayBio C-Series Human Cytokine Antibody Array C5 Decoder of cytokines in Figure 1C.

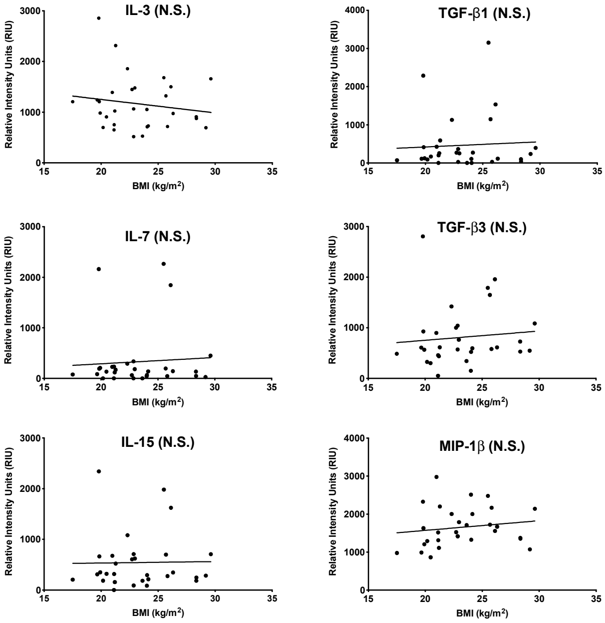

Supplemental Figure S3. Cytokine profiles that were significantly different with age or AMH but did not change with BMI. Fibroinflammatory markers IL-3, IL-7, IL-15, TGF-β1, TGF-β3, and MIP-1β relative intensity levels were not significantly correlated with BMI (kg/m2) in our patient population (total n=30) undergoing ART. N.S. = not statistically significant.

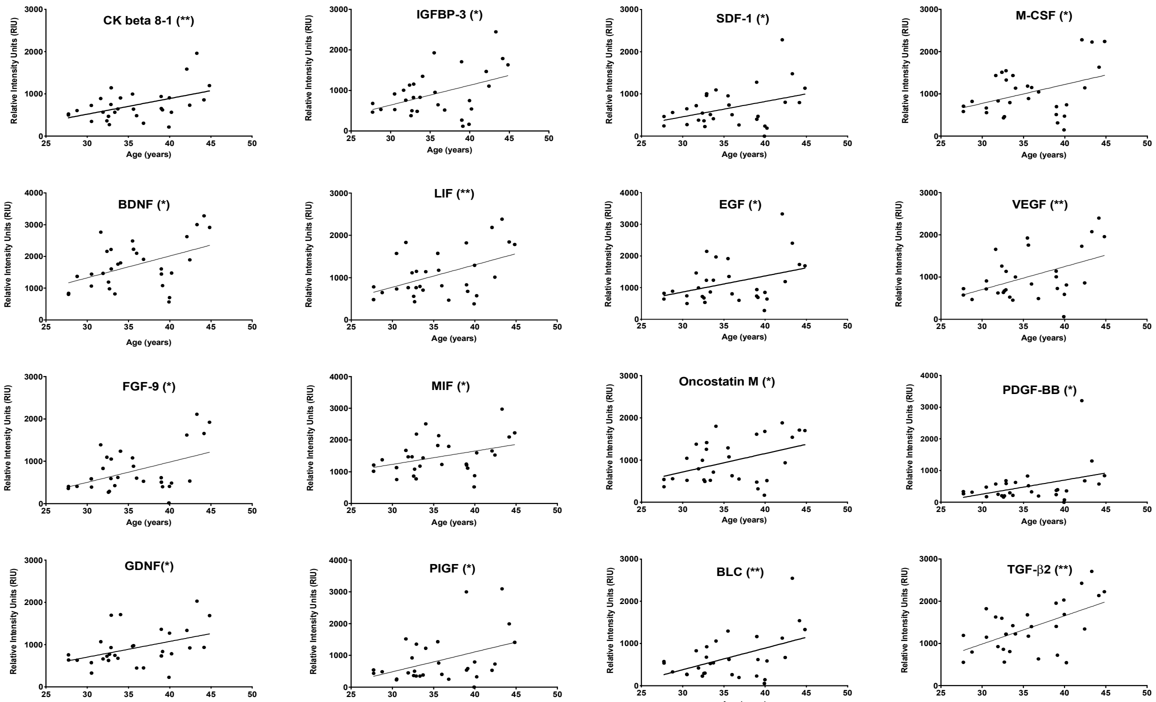

Supplemental Figure S4. Follicular fluid cytokine profiles that increased with chronologic age but not with reproductive age (AMH). Fibroinflammatory marker relative intensity levels exhibited a significant positive correlation with increasing chronological age (years) in n=30 patients undergoing ART. These cytokines have no significant correlation with AMH (ng/mL) levels. The lines correspond to the fitted regression equation. *P<0.05, **P<0.01.

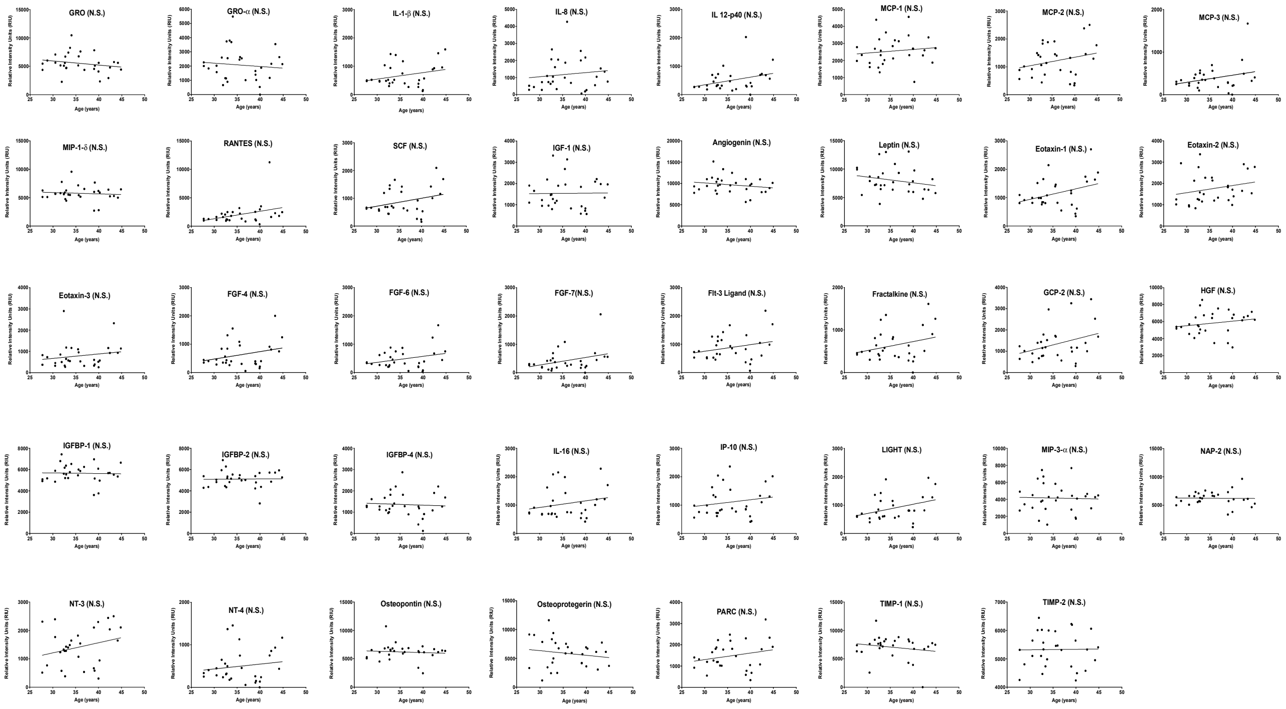

Supplemental Figure S5. Cytokine profiles in follicular fluid that showed no change with chronologic age. The lines correspond to the fitted regression equation. N.S. = not statistically significant.

Supplementary Table SI. Participant Cumulus Cell Information

Participant ID	Age	Diagnosis	Cumulus Cell Number	Oocyte Maturity Status	Embryo Outcome
Participant 31	37	Other Ovarian Failure	1	Mature	8 cell
			3	Mature	8 cell
Participant 32	32.5	Decreased Ovarian Reserve	1	Mature	Blastocyst
			2	Mature	Blastocyst
Participant 33	44.7	Unexplained	8	Mature	Blastocyst
			4	Mature	Blastocyst
Participant 34	33.9	Other	3	Mature	Hatching Blastocyst
			6	Mature	Hatching Blastocyst
			8	Mature	Hatching Blastocyst

Participant 35	41.5	Unexplained	1<br>2<br>3	Mature<br>Mature<br>Mature	Blastocyst<br>Blastocyst<br>Blastocyst
Participant 36	34.4	Other	4<br>5<br>6	Mature<br>Mature<br>Mature	Blastocyst<br>Blastocyst<br>Early Blastocyst
Participant 37	42.3	Decreased Ovarian Reserve	2<br>5<br>1	Mature<br>Mature<br>Mature	6 cell<br>Mature (no fert)<br>Early Blastocyst
Participant 38	34.8	Other	3<br>5<br>6	Mature<br>Mature<br>Mature	Blastocyst<br>Blastocyst<br>Blastocyst
Participant 39	40.1	Other	5<br>1<br>6	Mature<br>Mature<br>Mature	Blastocyst<br>Blastocyst<br>Blastocyst
Participant 40	34.9	Other Ovarian Dysfunction	1<br>2<br>6	Mature<br>Mature<br>Mature	Early Blastocyst<br>Early Blastocyst<br>Morula
Participant 41	43	Other	1<br>3<br>4	Mature<br>Mature<br>Mature	10 cell<br>6 cell<br>Morula
